# Fenofibrate promotes PPARα-targeted recovery of the intestinal epithelial barrier at the host-microbe interface in dogs with diabetes mellitus

**DOI:** 10.1038/s41598-021-92966-7

**Published:** 2021-06-29

**Authors:** Katti R. Crakes, Jully Pires, Nina Quach, Riley E. Ellis-Reis, Rachel Greathouse, Kathyrnne A. Chittum, Jörg M. Steiner, Patricia Pesavento, Stanley L. Marks, Satya Dandekar, Chen Gilor

**Affiliations:** 1 Department of Medical Microbiology and Immunology, School of Medicine, University of California Davis, Davis, CA 95616 USA; 2 Department of Medicine and Epidemiology, School of Veterinary Medicine, University of California Davis, Davis, CA 95616 USA; 3 Gastrointestinal Laboratory, Small Animal Clinical Sciences, College of Veterinary Medicine and Biomedical Sciences, Texas A&M University, College Station, TX 77843 USA; 4 Department of Pathology, Microbiology, and Immunology, School of Veterinary Medicine, University of California Davis, Davis, CA 95616 USA; 5 Present Address: Department of Small Animal Clinical Sciences, College of Veterinary Medicine, University of Florida, 2015 SW 16th Ave., Gainesville, FL 32610 USA

**Keywords:** Endocrine system and metabolic diseases, Experimental models of disease, Gastroenterology, Endocrine system and metabolic diseases, Gastrointestinal diseases, Metabolic disorders

## Abstract

Diabetes mellitus (DM) is associated with a dysfunctional intestinal barrier and an increased risk for systemic infection and inflammation in people, though the pathogenic mechanisms leading to this are poorly understood. Using a canine model of DM, we showed that the peroxisomal proliferator-activated receptor-α agonist fenofibrate modulates plasma lipid profiles and markers of intestinal barrier function. A 3-week course of fenofibrate reduced fasting interstitial glucose and inflammatory cytokine IL-8 and TNF-α concentrations, which correlated with reduced triglyceride levels. The lipidomic profile exhibited significantly lower levels of triacylglycerols, phosphatidylethanolamines, diacylglycerols, and ceramides following fenofibrate administration. On histopathological analysis, we observed an aberrant amount of intraepithelial CD3^+^ T lymphocytes (IEL) in the small intestine of dogs with spontaneous and induced-DM. Fenofibrate reduced IEL density in the duodenum of dogs with DM and enhanced markers of intestinal barrier function in vivo and in vitro*.* There were minimal changes in the intestinal microbial composition following fenofibrate administration, suggesting that repair of intestinal barriers can be achieved independently of the resident microbiota. Our findings indicate that lipid metabolism is critical to functionality of the intestinal epithelium, which can be rescued by PPARα activation in dogs with DM.

Diabetes mellitus and prediabetes increase the risk for other metabolic complications, such as obesity, cardiac disease, hypertension, and hyperlipidemia, collectively referred to as Metabolic Syndrome. Metabolic complications commonly coincide with gastrointestinal inflammation and increased intestinal permeability^1,2^. Disruption of the intestinal barrier can have detrimental consequences, including changes in intestinal microbiota, inflammatory cytokine production, increased bacterial translocation, signaling of toll-like receptors, and risk for gastrointestinal infections^1,3,4^. There is a great need for targeted therapies, but a critical gap exists between understanding the mechanistic link between metabolic disorders and gastrointestinal dysfunction^5^. We previously reported that peroxisome proliferator-activated receptor-alpha (PPARα) signaling, which regulates mitochondrial fatty acid beta-oxidation, may be key to restoring integrity of the intestinal barrier during chronic intestinal inflammation^6^. In this proof-of-concept study, we investigate the effects of fenofibrate, a PPARα agonist, on lipid profiles, systemic and intestinal inflammation, and intestinal barrier function in a canine DM model.

Highly expressed by hepatocytes, adipocytes, cardiomyocytes, enterocytes, and the renal cortex^7^, PPARα is recognized as a key regulator of metabolic pathways affected by DM. Deletion of PPARα in hepatocytes impairs fatty acid catabolism, increases circulating free fatty acids, and exacerbates non-alcoholic fatty liver disease^8^. By contrast, activation of PPARα markedly improves insulin sensitivity by increasing oxidation of fatty acids and minimizing lipotoxicity^9,10^. In human pancreatic islet cells, treatment with a PPARα agonist improves beta-cell responses and insulin secretion and prevents apoptosis and triglyceride accumulation^11^. In the clinical setting, fenofibrate is used to treat dyslipidemia, DM, and cardiovascular disease associated with metabolic syndrome^12^. It is unknown, however, whether activation of PPARα in vivo by oral dosing of fenofibrate can alleviate intestinal inflammation or reverse barrier disruption in patients with DM. Understanding the loss of intestinal barrier integrity and its relationship with intestinal microbiota in DM is critical to controlling intestinal inflammation and improving glycemic control^13^.

We utilized a canine model of DM to investigate both clinical and molecular outcomes of PPARα activation. Within 3 weeks of oral fenofibrate treatment, plasma lipids profiles were altered, which correlated with reduced systemic immune activation. Fenofibrate enhanced tight junction protein expression in the small intestine and reduced intraepithelial T lymphocyte numbers in the duodenum. Our data highlight the critical role of PPARα signaling and fatty acid metabolism in maintenance of gastrointestinal homeostasis and as a potential therapeutic target in DM.

## Research design and methods

### Animal study design and sample collection

All dogs involved in this study were provided by Sinclair Research LLC and housed under the care of both UC Davis Campus Veterinary Services (CVS) and Teaching and Research Animal Care Services (TRACS). This study was approved by the Institutional Animal Care and Use Committee (IACUC protocol #21100) and conduced in accordance with all applicable regulations and guidelines, including the ARRIVE 2.0 guidelines^14^. Seven male, 1.5-year-old beagles (weighing 7.3–11.7 kg) were housed individually with free access to drinking water and supervised access to outdoor play pens. Diabetes was induced with alloxan and streptozotocin, as previously described^15^, 8 months before this study. During the 8 weeks preceding the study, dogs were treated with ~ 0.7 U/kg porcine zinc insulin suspension (Vetsulin®, Merck Animal Health, Madison, NJ) at the time of their evening meal. The insulin dose was unchanged throughout the study. Using criteria for diagnosis and monitoring of DM as defined by the European Society for Veterinary Endocrinology in collaboration with the Society of Comparative Endocrinology^16^, all dogs were in good diabetic control as assessed by a board-certified veterinary internal medicine specialist (CG) as previously described^17^. Body weight and general health assessment were performed weekly and all dogs maintained their body weight and ideal body condition score with no evidence of gastrointestinal disease throughout this period. All dogs were monitored daily throughout the study and fed a commercial canine lab chow to meet their daily metabolizable energy requirement (300 g/day divided into 0700 h and 1900 h feedings). Dogs were administered fenofibrate (Tricor®, AbbVie Inc. North Chicago, Illinois, USA) at a dose of 10 mg/kg orally once daily for 3 weeks (days 1–21) as previously described^18^. Due to the nature of the pre-post study, randomization was not performed and personnel conducting the study were not blinded. In ethical consideration of reducing the number of animals used in this study, no control group (consisting of dogs that did not receive fenofibrate) were used.

Peripheral blood and fresh fecal samples (within 15 min of defecation) were obtained on days − 14 (pre-treatment baseline), 7 and 21 of fenofibrate administration. Blood was collected into EDTA and lithium heparin tubes. Fecal samples were collected, immediately frozen, and stored at  − 80 °C within 2 h of sample collection. Intestinal biopsies were obtained by video endoscopy on day  − 1. Animals were fasted prior to video endoscopy and two warm water enemas were administered six hours apart via a red rubber catheter on the day  − 2. On the morning of the endoscopy procedure, dogs were premedicated with acepromazine-butorphanol-atropine (0.02–0.04 mg/kg, 0.1–0.4 mg/kg, 0.02–0.04 mg/kg, respectively) and induced with diazepam (0.25–1 mg/kg) and ketamine (5–15 mg/kg). Endoscopy was performed to obtain multiple 2 mm mucosal pinch biopsies from the duodenum, ileum, and colon. Similar to the sample collection procedures at baseline, animals were fasted and received enemas on day 20. On day 21, animals were euthanized in accordance with the AVMA guidelines for the Euthanasia of Animals (2013).

### Flash glucose monitoring system (FGMS)

The FGMS sensor (Freestyle Libre, Abbott, UK) validated for use in dogs was utilized to measure interstitial glucose (IG) concentrations as previously described^19^. In brief, a single sensor was placed on a shaved area on the neck and used until sensor failure (10–14 days) and then a new sensor was placed, to achieve continuous measurement of IG from baseline until day 21. Accordingly, IG concentrations at each timepoint were averaged over 72 h (‘baseline’: days  − 7 to  − 5, ‘Day 7’: days 6–8, ‘Day 14’: days 13–15, ‘Day 21’: days 19–21). In addition, fasting IG was measured on days − 1 and 21 for 12 h starting at 1900 h (12 h after the last meal), when the evening meal was withheld, until the following meal was given.

### Plasma and serum markers

Concentrations of C-reactive protein (CRP), triglycerides, cytokines, and endotoxin were measured on days  − 14, 7, and 21. C-reactive protein was measured in lithium heparin-separated plasma on a Catalyst DX Chemistry Analyzer (IDEXX, USA) at the UCD VMTH. Serum triglycerides were measured at UCD Veterinary Clinical Laboratory Services. Plasma cytokines (IL-2, IL-6, IL-8, and TNF-α) were measured using a commercial multiplex electrochemiluminescence immunoassay (Meso Scale Diagnostics), as previously described^20^. Lipopolysaccharide (LPS) endotoxin was measured in plasma by Limulus Amoebocyte Lysate Pyrogent-5000 from Lonza (catalog #N384).

### Plasma lipidomics

Plasma was transferred to an ultra-high-pressure liquid chromatography column optimized for high retention and separation of lipid classes. Data processing was performed using MS-DIAL^21^, followed by a blank subtraction in Microsoft Excel and cleanup using MS-FLO. Peaks were annotated by manual comparison of MS/MS spectra and accurate masses of the precursor ion to spectra given in the LipidBlast spectral library from the West Coast Metabolomics Center at UCD, CA. Peak candidates were verified by MassHunter Quant software and analyzed by targeted MS/MS. Multivariate principal coordinate analysis was performed using MetaboAnalyst 4.0.

### Histopathology

Intestinal biopsies were obtained endoscopically on day 0 and full-thickness intestinal tissues were collected at necropsy. Tissues were fixed in 4% paraformaldehyde (PFA) for 24 h and paraffin-embedded. Five µm sections were stained with hematoxylin and eosin (UCD Anatomic Pathology Core) and were histologically evaluated. Blinded histological assessments were performed by a single board-certified veterinary pathologist (PP) in accordance to published histological standards^22^.

In addition, a retrospective study of canine DM was completed using archived samples from the pathology service at the UC Davis (UCD) Veterinary Medical Teaching Hospital (VMTH). The hospital’s electronic database was utilized to identify cases of client-owned dogs diagnosed with DM that died or were euthanized between 1990 and 2020. Selection criteria included cases with a definitive diagnosis of DM in the absence of other concurrent diseases (with the exception of pancreatitis). Tissue biopsies from the small intestinal of 9 patients were identified and subsequently evaluated histologically.

### Immunohistochemistry and confocal microscopy

Deparaffinized tissue sections were hydrated in a graded series of ethanol, blocked, and incubated with the following primary antibodies: rabbit polyclonal anti-claudin-1 (Invitrogen, cat #71-7800), mouse monoclonal anti-CD3 (DAKO, cat #M7254), and mouse anti-e-cadherin (BD Biosciences, cat #10182). Tissues stained with anti-claudin-1 and CD3 antibodies were incubated with the following secondary antibodies: Alexa Fluor 488 donkey anti-rabbit, Alexa Fluor 555 donkey anti-mouse (Invitrogen, Carlsbad, CA) or incubated with streptavidin-HRP for diaminobenzidine stains. Nuclei were visualized using DAPI nucleic acid stain (Invitrogen) and slides were mounted using 20 μl ProLong Gold Antifade Mountant (Thermo Fisher, Waltham, MA).

Confocal z-stacks were captured using a Leica SP8 STED 3 × confocal microscope (Leica Microsystems, Germany) with a white light laser. A 63x/1.4NA oil immersion objective and maximal image size of 1248 × 1248 pixels were utilized for all acquisitions. Z-stacks were performed with a 0.3 μm step size at 1.25 × capturing full thickness of individual channels. Four images representative of each tissue section were captured for quantitative analysis. All confocal images were analyzed on Imaris Version 8.2 for 3-D visualization. A threshold was applied to all images to eliminate background fluorescence. Sum fluorescence intensities and volume were calculated from the chosen regions of interest, as demarcated from the apical end of the epithelial cell nuclei to the edge of the brush border.

### Cell cultures

Caco-2 (ATCC® HTB-37) cells were purchased from ATCC and routinely grown at 37ºC in MEM tissue culture medium supplemented with 10% FBS and 1% Anti-Anti (Thermo Fisher). Caco-2 cells were sub-cultured after partial digestion with 0.05% trypsin–EDTA and cells from passages 10–20 were used for subsequent analysis. Cells were seeded onto 8-well chamber slides (Thermo Fisher) and allowed to grow for 48 h to form confluent monolayers prior to treatment with 10 μM fenofibrate (Sigma), 3 μM GW6471 (Sigma), recombinant TNF-α (PeproTech) and D-glucose (Gibco). Caco-2 cells were fixed in 4% paraformaldehyde, blocked in 15% donkey serum in PBS, and incubated in 1:50 rabbit anti-human ZO-1 (Invitrogen) antibody overnight, followed by donkey anti-rabbit Alexa Fluor 488 for 1 h. Glass coverslips were mounted using Prolong Diamond Anti-Fade. Images were captured using the EVOS FL Auto Imaging System (Thermo Fisher). Cell images were segmented using Morphological Segmentation ImageJ plug-in and junction tortuosity was calculated as a ratio between junctional length and Euclidian distance between two points, as previously described^23^. For gene knockdown studies, Caco-2 cells were seeded in a 12-well plate and transfected with PPARα siRNA (AM51331, Thermo Fisher) or nontargeting control siRNA (AM4611). Cells were treated overnight and lysed for quantitative reverse transcriptase PCR (qRT-PCR) of PPARα gene expression, as previously described^6^.

### Intestinal microbiota analysis by 16S sequencing

Intestinal microbiota composition in fecal samples was assessed using 16S rRNA sequencing as previously described^24^. DNA was isolated using the Qiagen DNeasy PowerSoil kit (Qiagen). Individual amplicons were pooled in equal concentrations and cleaned using Ampure XP beads (Beckman Coulter). The band of interest was subjected further to isolation via gel electrophoresis on a 1.5% Blue Pippin HT gel (Sage Science). The library was quantified using qPCR followed by 300-bp paired-end sequencing using Illumina MiSeq in the Genome Center DNA Technologies Core, University of California, Davis. Demultiplexing, removal of chimeras, rarification, and quality filter of low-abundance sequencing reads were performed by the UC Davis Host Microbe Systems Biology Core Facility. Analysis of alpha and beta-diversity were performed on R software^25^. Differential abundances were analyzed using DESeq2 with phlyoseq and omu on R^26–28^.

### Statistical analysis

Graphpad Prism v9 was used for statistical analyses and data plotting. Significant differences between 2 paired samples were analyzed using a Wilcoxon matched-pairs signed rank test and comparisons between 3 or more paired samples were analyzed using the Friedman test with a post-hoc Dunn’s multiple comparisons test. Increases and decreases in the plasma lipidome were determined when *p* < 0.05 and fold-change was greater than 1.5×. Fluorescence values from immunohistochemistry were analyzed using a mixed model ANOVA to incorporate 4 values for each tissue section into the statistical model. In all tests, significance was set at *p* < 0.05.

### Data and resource availability

The sequences and metadata reported in this paper have been deposited in NCBI Bioproject at https://www.ncbi.nlm.nih.gov/bioproject (PRJNA667294).

## Results

### Reduction of fasting glucose levels and serum triglyceride concentrations is associated with decreased immune activation following fenofibrate treatment

**Figure 1:**
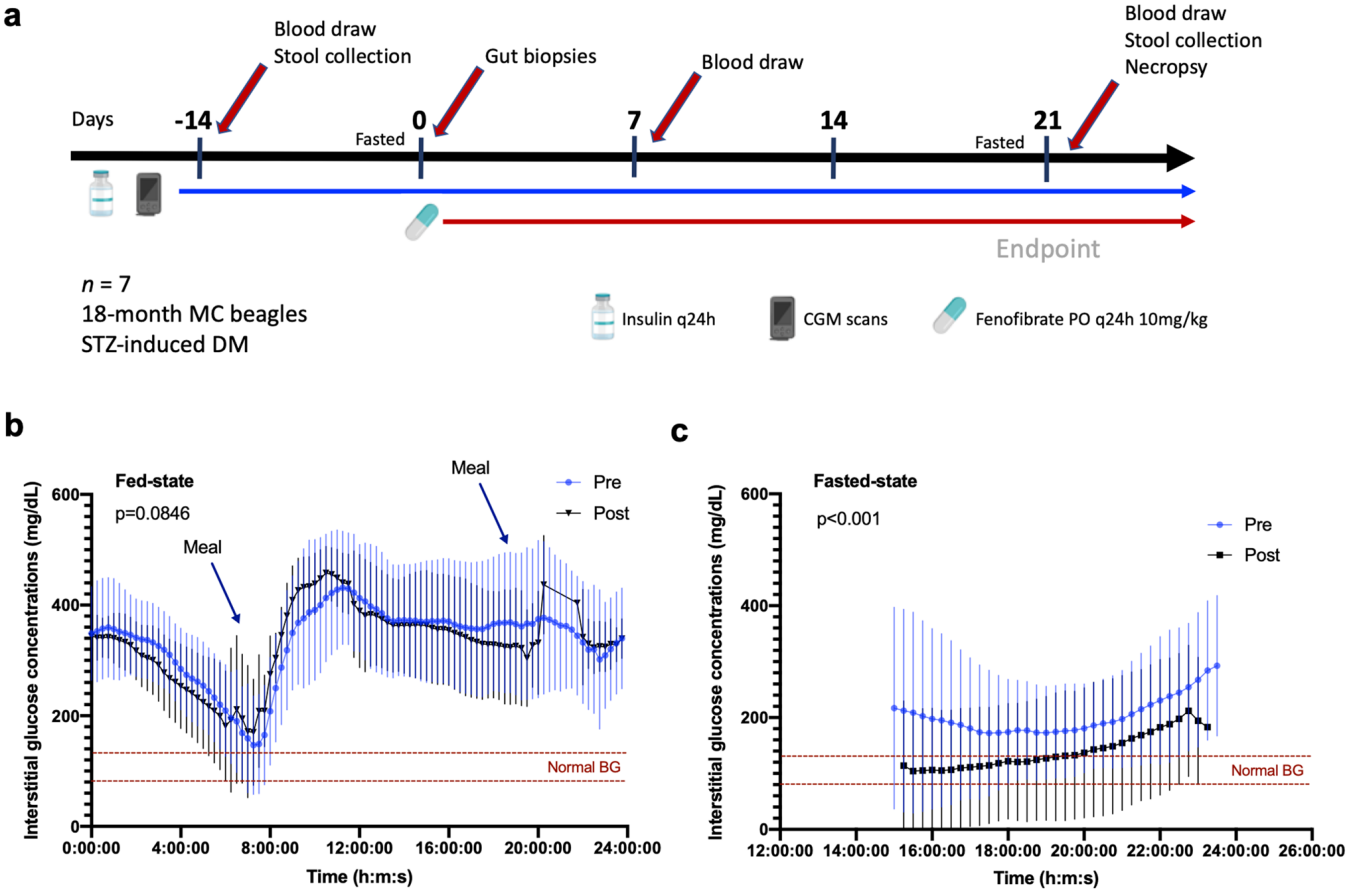
Fenofibrate reduces fasting interstitial glucose (IG) concentrations in diabetic dogs. (**a**) Diabetic dogs treated with fenofibrate (10 mg/kg by mouth) for 21 days. (**b**) Mean IG measurements over 72 h after 3 weeks of fenofibrate administration compared to pre-treatment concentrations. (**c**) Comparison of fasted mean IG measurements after fenofibrate administration compared to baseline concentrations. All data represent the average of 7 dogs evaluated in 15-min increments. Means ± SD are plotted. *P*-values were determined by the Wilcoxon test. Image icons were created with BioRender.

**Figure 2:**
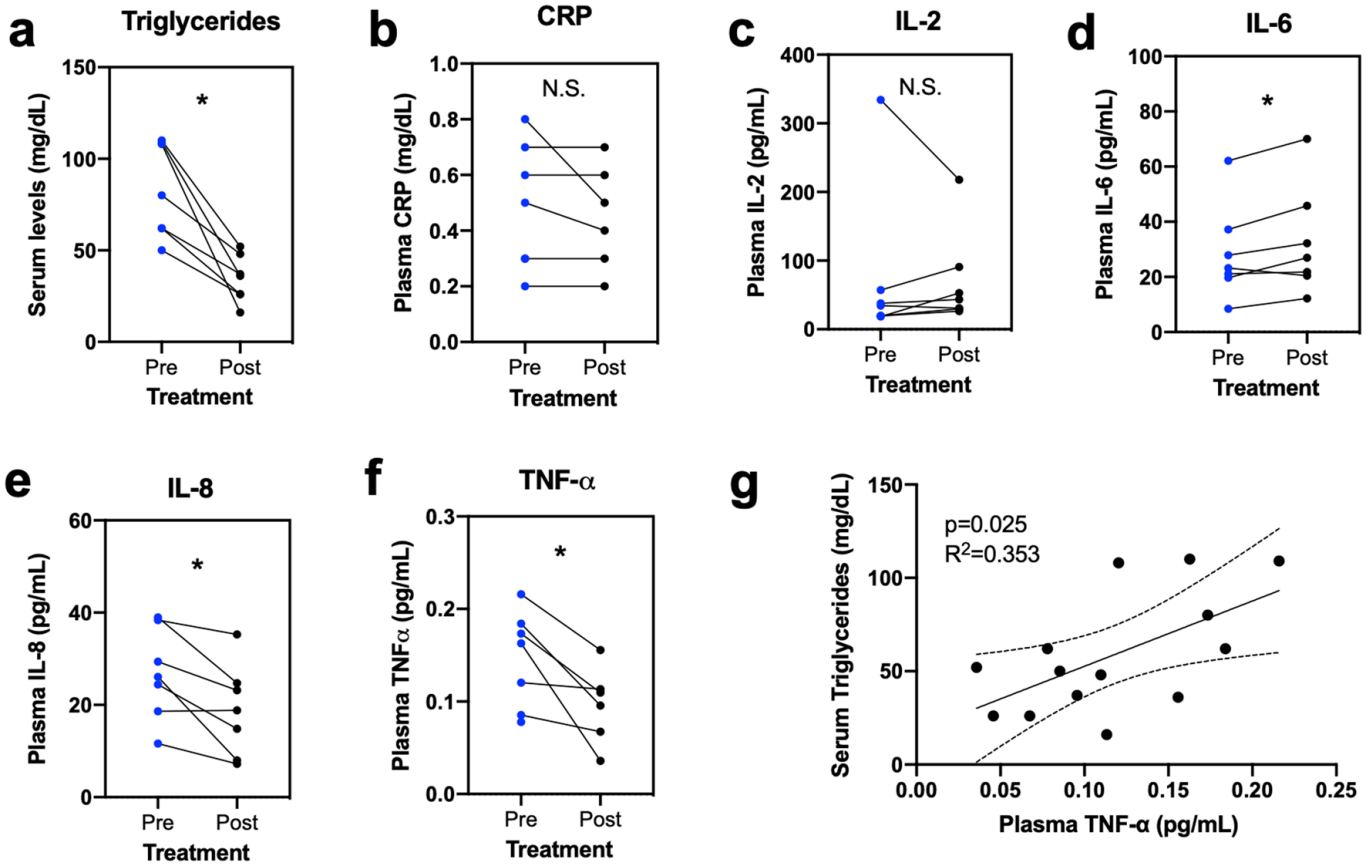
Fenofibrate significantly lowers serum triglyceride concentrations and plasma IL-8 and TNF-α concentrations. (**a**) Serum triglyceride concentrations in 7 diabetic dogs treated with fenofibrate for 3 weeks (Day 21) compared to concentrations at baseline (Day −14). Comparison of plasma (**b**) CRP and inflammatory cytokines (**c**) IL-2, (**d**) IL-6, (**e**) IL-8, and (**f**) TNF-α following fenofibrate administration. (**g**) Correlation of serum triglyceride concentrations and plasma TNF-α concentrations. *P < 0.05, **P < 0.01 by Wilcoxon test. N.S., not significant.

To achieve our objective of assessing whether fenofibrate reduces triglyceride levels and hyperglycemia, we administered fenofibrate to seven diabetic beagles daily and collected samples throughout the course of the study (Fig. 1a). At baseline, interstitial glucose (IG) concentrations were high in dogs with DM, reaching a nadir of approximately 146 mg/dL after insulin administration^29^ (Fig. 1b). We measured 72 h mean IG concentrations after 3 weeks of fenofibrate treatment and observed no changes in IG compared to pre-treatment baseline (*p* = 0.0846), indicating minimal changes in daily glycemic control. Only small changes in IG concentrations were observed at Day 7 (*p* < 0.0001, rank sum diff = 118.0) compared to baseline levels (Supplementary Fig. S1). In dogs, the post-prandial absorptive phase often lasts 6–9 h indicating that these 72 h IG data mostly represent the fed state with q12 feeding periods^30–32^. To assess the effect of fenofibrate in the fasting state, we compared the mean 10 h IG, as measured between 14–24 h after the previous meal (when the evening meal was skipped in preparation for next day procedure). The mean 10 h fasting IG was lower (*p* < 0.0001) after 3 weeks of oral fenofibrate (Fig. 1c) compared to baseline. In addition, serum triglyceride concentrations were reduced on day 21 compared to baseline (Fig. 2a). Plasma C-reactive protein (CRP) concentrations were within the reference interval at baseline and did not change after 21 days of treatment (Fig. 2b). On day 21, no significant changes in IL-2 concentrations were observed (Fig. 2c). However, fenofibrate administration was associated with an increase in IL-6 concentration (mean ± SD: 32.8 ± 19.5 pg/mL) compared to baseline (28.6 ± 17.2 pg/mL, *p* = 0.0469) and a decrease in plasma IL-8 concentration (18.9 ± 9.9 pg/mL) compared to baseline (26.8 ± 9.9 pg/mL). Plasma concentrations of TNF-α were also significantly decreased (0.096 ± 0.041 pg/mL) compared to baseline (0.146 ± 0.052 pg/mL, p = 0.0312) (Fig. 2d–f), and correlated with serum triglyceride concentrations (*p* = 0.025) (Fig. 2g).

### PPARα activation shifts plasma lipid profiles in dogs with DM

**Figure 3:**
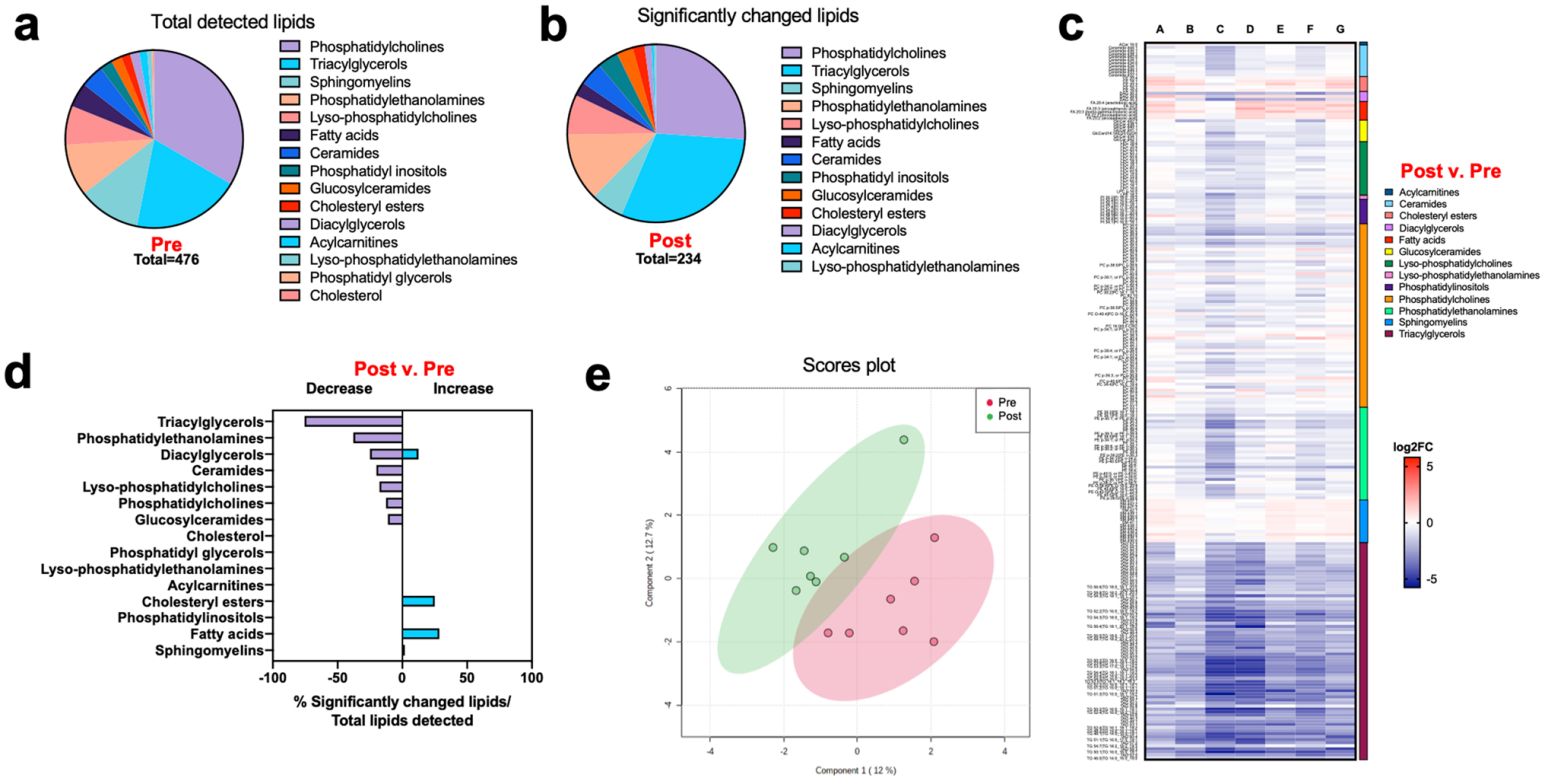
Fenofibrate alters lipid metabolism in diabetic dogs. (**a**) Composition of the plasma lipidome in 7 diabetic dogs at baseline, categorized by lipid groups. (**b**) Lipid groups are altered after 3 weeks of fenofibrate treatment. Significance determined when *P* < 0.05. (**c**) Heatmap of all differentially abundant lipid species in plasma of diabetic dogs pre- and post-fenofibrate treatment. (**d**) Percentage of lipid groups that were differentially abundant after fenofibrate administration. Significance determined when *P* < 0.05 and fold-change > 1.5. (**e**) Principal component analysis of the plasma lipidome in diabetic dogs pre- and post-fenofibrate administration. Circles represent regions of 95% confidence.

To determine the effects of fenofibrate on cellular metabolism and systemic lipid composition, we assessed the plasma lipidome at 3 weeks post-fenofibrate treatment compared to the composition at baseline. At baseline, a total of 476 unique lipids were detected in all samples, 33% of which were phosphatidylcholines, 20% triacylglycerols, 11% sphingomyelins, 9% phosphatidylethanolamines, and 7% lyso-phosphatidylcholines (Fig. 3a). At Day 21 of fenofibrate administration, we observed a shift in lipid composition consisting of 234 altered lipids out of the total 476 detected (Fig. 3b). There was some variability between dogs, but changes in lipid groups were consistent across all individuals (Fig. 3c). Of the detected lipids, 75% triacylglycerols, 38% phosphatidylethanolamines, 25% diacylglycerols, and 20% ceramides were decreased, whereas 28% fatty acids and 25% cholesteryl esters were increased (Fig. 3d). Principal coordinate analysis of plasma lipidome analysis revealed that samples collected on day 21 did not cluster with samples from baseline (Fig. 3e).

We also evaluated whether changes in the plasma lipidome would occur within a short duration (7 days) after fenofibrate treatment. Plasma lipidomic analysis revealed only 138 lipids that were significantly changed compared to baseline concentrations (Supplementary Fig. S2a). Significant changes were also relatively consistent across all individuals (Supplementary Fig. S2b). Of all detected lipids, 100% acylcarnitines, 86% fatty acids, 50% lyso-phosphatidylethanolamines, and 20% ceramides were decreased after 7 days of fenofibrate administration (Supplementary Fig. S2c). Principal coordinate analysis revealed separation between the two groups with some overlap between samples collected on day 7 and baseline (Supplementary Fig. S2d). Although some lipid families were significantly altered with fenofibrate on both Day 7 and Day 21, many exhibited a different pattern, indicating a time-dependent response with an early effect on acylcarnitines and fatty acids at Day 7 and a later effect on triacylglycerols at Day 21 (Supplementary Fig. S2e).

### Fenofibrate treatment enhances markers of intestinal barrier function during DM

**Figure 4:**
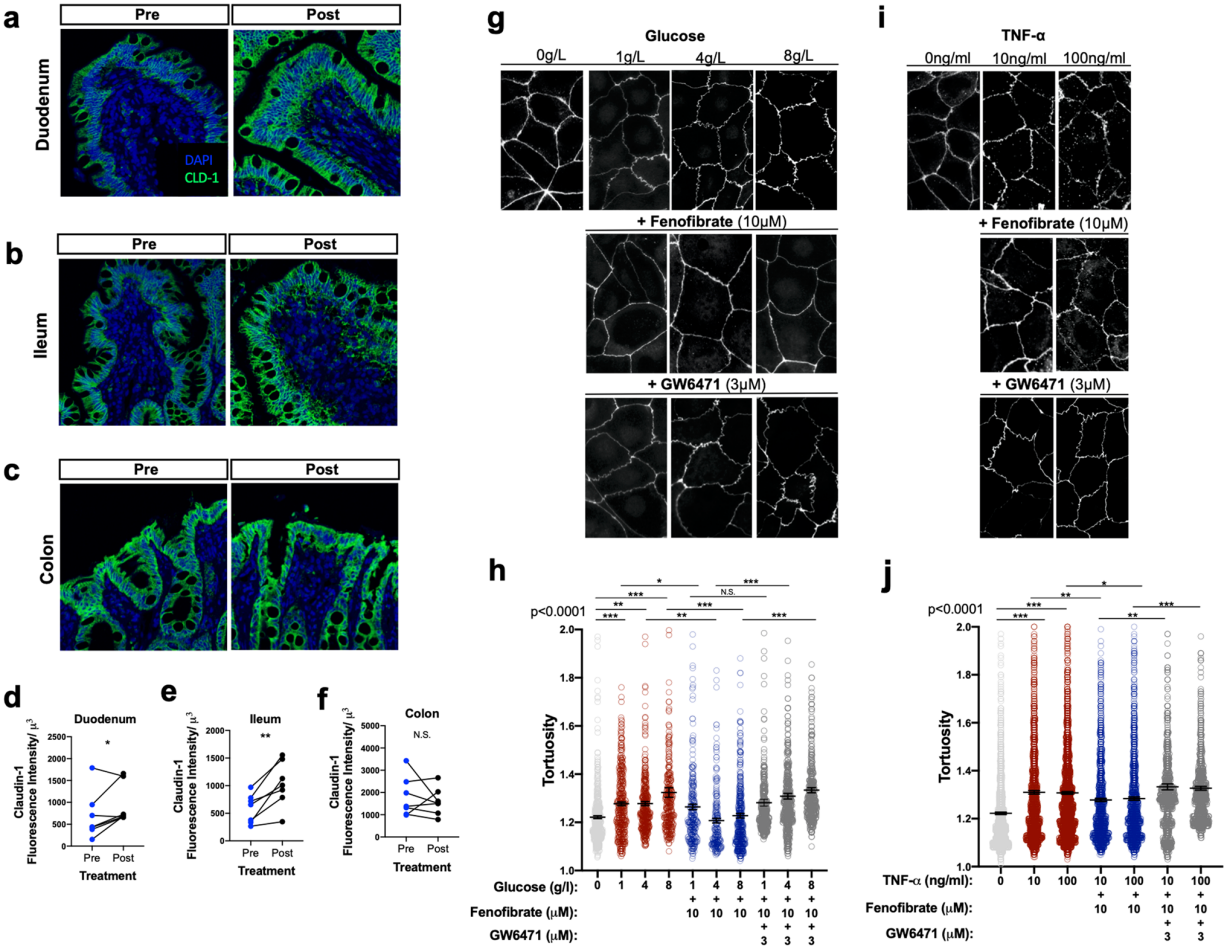
Fenofibrate upregulates expression of tight junctions on intestinal epithelial cells. Immunofluorescent staining of claudin-1 (CLD-1) expression in the (**a**) duodenum, (**b**) ileum, and (**c**) colon of diabetic dogs pre- and post-fenofibrate administration. (**d**–**f**) Semi-quantitation of CLD-1 protein expression in the intestinal compartments. (**g**) Representative ZO-1 staining and (**h**) quantification of barrier tortuosity in Caco-2 cells treated with different concentrations of glucose + 10 μM fenofibrate + 3 μM GW6741 or (**i**, **j**) TNF-α + fenofibrate + GW6741 for 24 h. *P < 0.05, **P < 0.01, ***P < 0.001 by (**a**–**f**) mixed models ANOVA and (**g**–**j**) one-way ANOVA with Tukey’s multiple comparison test. N.S., not significant.

We explored whether intestinal barrier disruption in canine DM could be targeted for repair with fenofibrate treatment in vivo*.* Given the complexity of junctional complexes and regulation of intestinal permeability, we measured claudin-1 and e-cadherin expression as a proxy for intestinal barrier integrity, which when downregulated, leads to increased intestinal barrier permeability through NF-κB activation^33^. Treatment with fenofibrate for 3 weeks increased structural integrity and expression of claudin-1 in the duodenum and ileum (Fig. 4a,b), but not the colon (Fig. 4c). Claudin-1 expression was enhanced (*p* = 0.0232, diff ± SE = 261.4 ± 110.9) in the duodenal epithelium (Fig. 4a), as indicated by the quantification of claudin-1 fluorescence intensity (Fig. 4d). Fenofibrate also enhanced claudin-1 expression in the ileum (*p* = 0.0003, diff ± SE = 446.5 ± 112.6) after fenofibrate administration (Fig. 4b,e). Similar changes were detected in e-cadherin expression, a key molecule in formation of adherence junctions and desmosomes (Supplementary Fig. S3). However, fenofibrate was not associated with changes in tight junction expression (*p* = 0.3009, diff ± SE =  − 204.6 ± 195.3) in the colonic epithelium (Fig. 4c,f).

To test whether fenofibrate alters the intestinal barrier through activation of PPARα, we utilized Caco-2 intestinal epithelial cells in PPARα-specific antagonism and gene knockdown. A significant reduction of PPARα expression was detected in PPARα siRNA-treated Caco-2 cells, which was not inducible following fenofibrate treatment (Supplementary Fig. S4). We then exposed Caco-2 intestinal cells to varying concentrations of glucose or TNF-α to model disruption of the intestinal barrier. Caco-2 cells were concurrently treated with fenofibrate and PPARα antagonist GW6471 to determine whether PPARα activation reverses epithelial barrier defects induced by glucose or TNF-α. As expected^34^, increasing concentrations of glucose induced barrier defects as measured by ZO-1 tortuosity (Fig. 4g). Cells treated with fenofibrate showed reduced barrier tortuosity compared to DMSO controls, which was inhibited by GW6471 (Fig. 4h). Similarly, increasing concentrations of TNF-α in the culture medium induced epithelial barrier defects (Fig. 4i), which was reversed following treatment with fenofibrate (Fig. 4j).

### Increased intraepithelial T lymphocyte density in the duodenum associated with DM is diminished after fenofibrate administration

**Figure 5:**
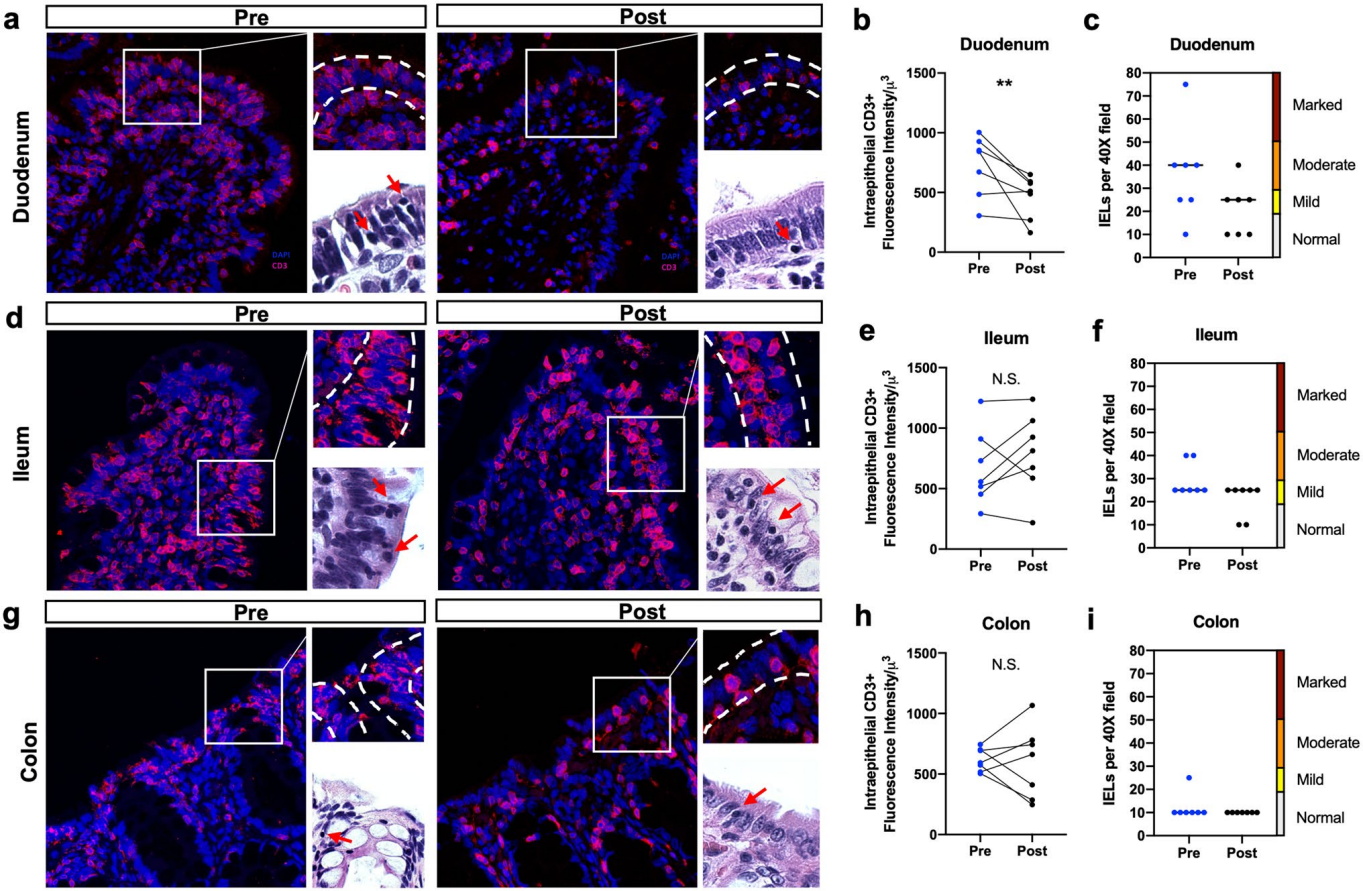
Fenofibrate reduces the density of intestinal intraepithelial CD3^+^ lymphocytes in diabetic dogs. (**a**) Immunohistochemical staining, (**b**) semi-quantitative assessment of CD3^+^ expression, and (**c**) scoring system for intraepithelial lymphocytes before and after fenofibrate administration. Staining, semi-quantitative assessment of CD3^+^ expression, and scoring system of intraepithelial lymphocytes in the (**d**–**f)** ileum and (**g**–**i**) colon. H&E staining of intraepithelial lymphocytes *(*bottom right*, red arrows*) in each compartment, 100×. *P < 0.05, **P < 0.01 by mixed models ANOVA. N.S., not significant.

We used histopathology to characterize the cellular and microstructural changes associated with fenofibrate administration in dogs with DM. Biopsies obtained during the study were examined for lacteal dilation, crypt/villus ratio, villus shape, epithelial injury, crypt dilation, and presence of lymphocytes, plasma cells, eosinophils, and neutrophils in the lamina propria. Most strikingly, the number of intraepithelial CD3^+^ lymphocytes (IELs) in the duodenum was mild to moderately increased (Fig. 5a, *red arrows*) at baseline^35^, and decreased after a 3-week course of fenofibrate (*p* = 0.002, diff ± SE =  − 263.0 ± 65.19) (Fig. 5b,c). Intraepithelial CD3^+^ lymphocytes infiltration was also observed in the ileum, but did not change following administration of fenofibrate (Fig. 5d–f). In addition, no changes in IELs were observed in the colon (Fig. 5g–i). To assess whether increased abundance of IELs was an anomaly to the canine cohort in our study, we performed a limited retrospective histopathological study on 9 client-owned dogs with spontaneous DM at the UCD VMTH that had been autopsied. Seven of these 9 dogs exhibited moderate-to-severe increases in IEL density in the small intestine, similar to findings in our purpose-bred dogs with experimentally induced DM (Supplementary Fig. S5).

### Intestinal microbiota composition remains unaltered following fenofibrate administration

**Figure 6:**
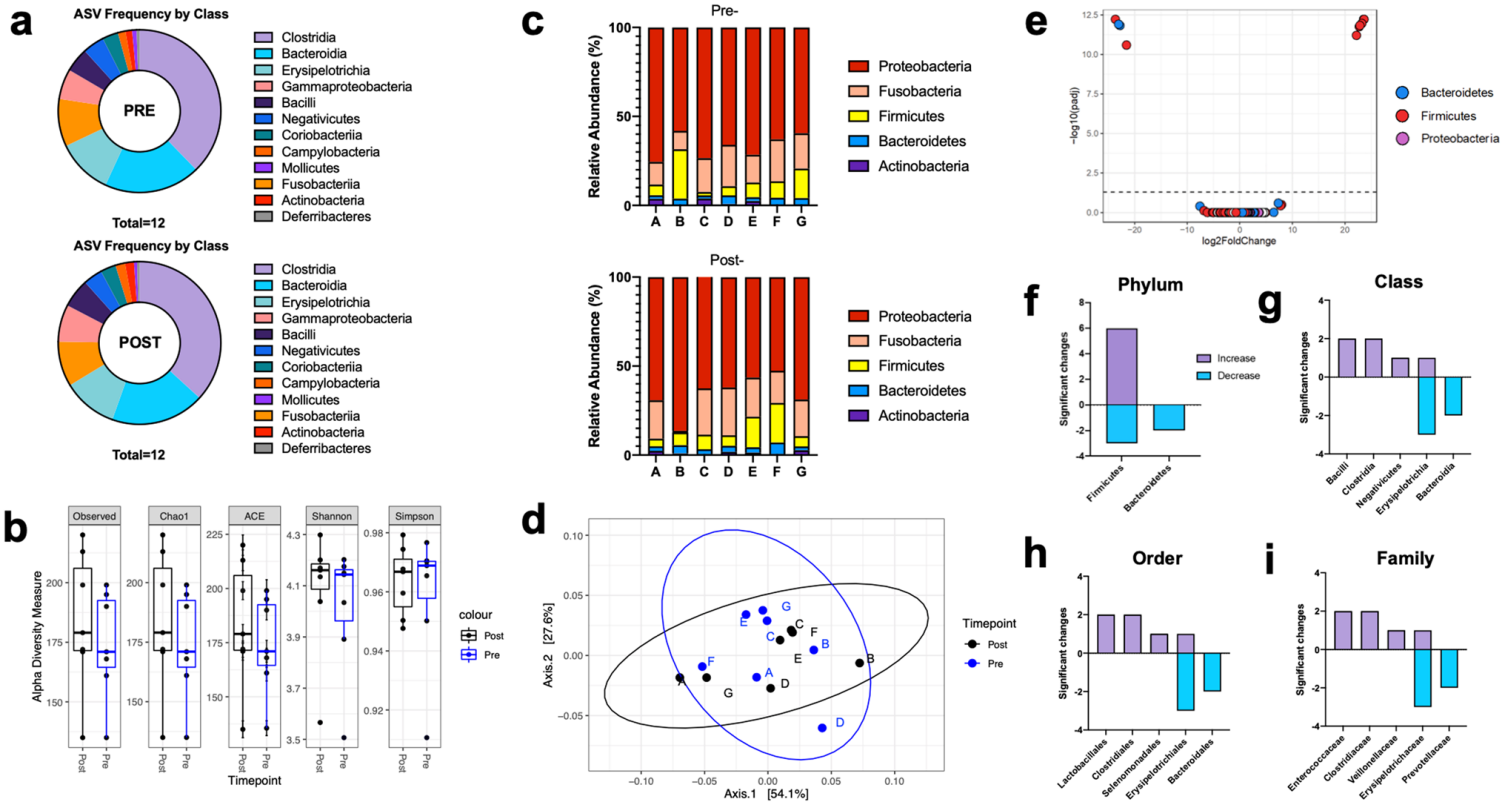
No changes in the fecal microbiota were associated with fenofibrate treatment in diabetic dogs. (**a**) Frequencies of amplicon sequence variants (ASVs) in fecal samples collected pre- and post-fenofibrate treatment. (**b**) Evaluation of alpha diversity using Observed, Chao1, ACE, Shannon, and Simpson indices. (**c**) Bar chart showing relative abundance of bacterial phyla among samples collected before and after fenofibrate administration in each of 7 dogs (*A-G)* with experimentally-induced DM. (**d**) Weighted UniFrac PCoA analysis of microbiota composition in identical animals pre- and post-fenofibrate treatment. Circles represent regions of 95% confidence. (**e**) Volcano plot representing differentially abundant bacterial phyla where *P* < 0.05. Numbers of differentially abundant bacterial (**f**) phyla, (**g**) class, (**h**) order, and (**i**) family between pre- and post-fenofibrate samples DESeq2 with *phyloseq* and *omu* on R software.

To investigate whether histopathological changes were associated with alterations in intestinal microbiota following fenofibrate administration, we collected fecal samples to assess the composition of the intestinal microbiota in dogs with experimentally induced DM after fenofibrate administration compared to that at baseline. Considering both sequence similarity and abundance within our samples^36^, we compared amplicon sequence variants (ASV) by bacterial class in all samples and found a predominance of 12 classes, including Clostridia, Bacteroidia, Erysipelotrichia, Fusobacteria, and Gammaproteobacteria (Fig. 6a). Negligible changes in ASV frequencies were observed in samples collected after fenofibrate treatment. Alpha diversity of samples revealed no differences following fenofibrate administration (Fig. 6b). Taxonomic evaluation of relative abundances at the level of bacterial phyla revealed animal-to-animal variation of the fecal microbiota, which remained stable after fenofibrate administration (Fig. 6c). This was consistent with the principal coordinate analysis of all samples, which showed clustering of samples before and after fenofibrate administration (Fig. 6d). Eleven OTUs were altered in samples after fenofibrate administration compared to baseline (*p* < 0.05) (Fig. 6e), which belonged to bacterial phyla Firmicutes and Bacteroidetes (Fig. 6f). Out of 742 unique OTUs detected in our samples, the 11 changed OTUs were mapped to minor changes at the level of bacterial class, order, and family (Fig. 6g–i).

## Discussion

New strategies to coordinate repair of the intestinal barrier and metabolic dysfunction are essential to combat systemic inflammation associated with DM. It is well-established that the intestinal barrier greatly influences disease pathogenesis^37^, but whether intestinal barrier disruption is the cause or consequence of diseases remains unclear^38^. In addition, it is unknown why the epithelial barrier fails to be restored in pathological conditions in spite of an abundant supply of proliferating epithelial stem cells. Accumulating evidence suggests that some answers may be uncovered by looking deeper into the host-microbiome interactions that regulate nutrition, immunity, and inflammation^39^. Understanding these networks might help identify post-biotic therapeutic targets such as PPARα. Beneficial intestinal microbes such as *Lactobacillus plantarum* activate PPARα, leading to improved intestinal barrier function^6^. Similar beneficial gut microbes have been shown to improve metabolic status in obesity and DM^40^, which may also involve PPARα signaling. Using a canine model of DM, we tested the effects of PPARα activation by fenofibrate and investigated gut mucosal and systemic responses after 3 weeks of treatment. This canine model allowed us to collect biological samples before and after treatment, eliminating genetic variability and diet as confounders, and greatly minimizing environmental confounders. Because canine DM bears many similarities to human type 1 DM, studies in dogs are invaluable for understanding pathogenesis and testing interventions for management of hyperglycemia, hyperlipidemia, and their consequences^41,42^.

Our study identified several mechanisms by which fenofibrate administration might alleviate gut-associated and systemic consequences of DM. First, fenofibrate reduced fasting interstitial glucose concentrations to normal blood glucose reference ranges. Reduction in fasting blood glucose concentrations are generally associated with a reduced risk for cardiovascular diseases, neuropathies, and diabetic nephropathy^43,44^. Secondly, fenofibrate enhanced epithelial tight junction protein expression in the small intestines. Studies show that hyperglycemia contributes to intestinal barrier disruption, leading to an influx of microbial pathogens and products that exacerbate inflammation^34^. We previously reported that PPARα activation by beneficial microbes may restore intestinal barrier function during chronic inflammation in rhesus macaques by rescuing mitochondrial function^6^. Combined with the in vivo findings in canine DM and in vitro findings on human epithelial cell cultures, our results point to PPARα agonists as a potential therapeutic target in the prevention and amelioration of intestinal barrier dysfunction and mucosal inflammation. Lastly, fenofibrate administration reduced the numbers of intraepithelial T lymphocytes in the duodenum during DM. Histopathological evaluation showed a high IEL density in dogs with DM in our study. The pathogenesis of IEL infiltration in diabetic dogs is not clear, but has been linked to malabsorption of nutrients and small bowel mucosal injury^45^, as we had observed in our study. Functional losses in intestinal barrier integrity may trigger autoimmune type 1 DM through activation of self-reactive T cells^46^, which could contribute to an increase in CD3^+^ IELs.

Changes in lipid metabolism may precede and exacerbate the development of DM^47,48^. Assessment of the lipid metabolism in patients with type 1 DM and healthy controls revealed changes in phosphatidylcholines, lyso-phosphotidylcholines, and sphingomyelins^49,50^, which may be associated with genetic polymorphisms in PPARs^51^. In our study, a 3-week course of fenofibrate altered triacylglycerols, phophatidylethanolamines, diacylglycerols, ceramides, lyso-phosphatidylcholines, phophatidylcholines, and glucosylceramides. We observed time-dependent patterns in lipid profiles after fenofibrate administration. At Day 7, acylcarnitines and fatty acids were significantly decreased from baseline, whereas triglyceride levels were minimally affected. At Day 21, triglycerides were significantly reduced with slight increases in cholesteryl esters and fatty acids. These findings painted a more complicated picture of lipid metabolism because we expected detectable lipids to decrease with a longer duration of fenofibrate administration. Since triglycerides were significantly decreased at this time point, we attributed the presence of cholesteryl esters to high-density lipoprotein (HDL) origin, which is beneficial for endothelial function, cardiovascular disease, and various lipid disorders^52^. Furthermore, 6 out of 21 fatty acids were mildly increased at Day 21, which may have been transient as a result of lipolysis or released during a prolonged fasting period. PPARα agonists such as fenofibrate rapidly increase the breakdown of fatty acids through beta-oxidation, forming fatty acylcarnitines and acetyl CoA within 1 week. Fenofibrate can also influence lipolysis, the breakdown of triglycerides into fatty acids and glycerol, but lowering of triglycerides may take up to 3 weeks. Changes in metabolomic profiles and fatty acids seen in canine DM differ from healthy control dogs and parallels that in human type 1 DM patients^53^, underscoring the value of investigating metabolic markers in dogs with DM. However, it is important to note that by virtue of being insulin-deficient, even with regular insulin treatment, dogs may not be able to achieve normal lipid profiles following treatment with fenofibrate.

It is unknown whether fenofibrate treatment decreases immune activation in DM by acting directly on lipid metabolism, intestinal barriers, or both. Evidence suggests that fenofibrate may have dual effects, enhancing fatty acid oxidation and directly preventing intestinal damage^54,55^. Our findings show that fenofibrate (1) reduced circulating levels IL-8 and TNF-α, which may alleviate insult to the intestinal barrier, and (2) increased circulating levels of IL-6, which may be advantageous in DM through stimulation of lipolysis, beta-oxidation, and leptin secretion^56^. It is still unclear why IL-6 was mildly elevated after fenofibrate administration, though we hypothesize that changes in lipid metabolism and fat storage may influence IL-6 expression independently of inflammatory cytokines TNF-α, IL-1, and IL-4. What has remained clear from our study and others is that regulation of intestinal barrier function is intimately connected to metabolism and that both must be considered in the development of new therapeutic targets^57,58^. For example, PPARs may play a role in repressing inflammatory responses through inhibiting NF-kB, macrophage polarization towards the anti-inflammatory M2 phenotype, and enhancing Th2 polarization in T cells^59^, which have yet to be studied in animal models of DM. Since there were negligible changes in fecal microbiota following fenofibrate administration, fenofibrate may have a predominantly host-mediated effect on intestinal barrier function. This could be, in part, due to the lack of difference in the fecal microbiota observed between diabetic and healthy dogs^60^. The objectives of our study were to investigate the effects of fenofibrate on intestinal health and metabolism in DM, so we did not include a healthy cohort kept at the same environmental conditions. Future longitudinal studies are warranted to investigate the effects of PPARα agonists on DM compared to a healthy cohort.

Collectively, our findings present associations between metabolic and intestinal mucosal health using the PPARα agonist, fenofibrate. We observed that fenofibrate improves plasma lipid profiles, markers of intestinal barrier integrity, and mucosal inflammation associated with DM. Further investigation into this canine model of DM is critical in discovery of new molecular pathways and development of novel therapeutic approaches in humans with metabolic or gastrointestinal disease^61,62^. The role of IELs in pathophysiology of canine diabetes mellitus remains under-investigated and warrants further research to identify underlying mechanisms that contribute or protect against intestinal barrier function. Currently, there are many limitations to in vivo intestinal barrier measurements in dogs, resulting in a need for more quantitative markers of barrier permeability and reliable in vitro models in dogs. More longitudinal studies are needed with a larger sample size consisting of both male and female dogs to confirm the findings reported herein. Our data highlight opportunities to explore PPARα signaling as a modulator of metabolism and mucosal immunity across multiple diseases and species. Based on these findings, clinical studies in humans with DM are warranted.

## Supplementary Information

Supplementary Information.
