# Supplementary material for: Fenofibrate promotes PPARα-targeted recovery of the intestinal epithelial barrier at the host-microbe interface in dogs with diabetes mellitus
Source: Sci Rep. 2021 Jun 29;11:13454. doi: 10.1038/s41598-021-92966-7 (PMC8241862; doi:10.1038/s41598-021-92966-7)
Supplement: Supplementary file 1 — Supplementary Information. [file 41598_2021_92966_MOESM1_ESM.pdf]

Supplementary Materials

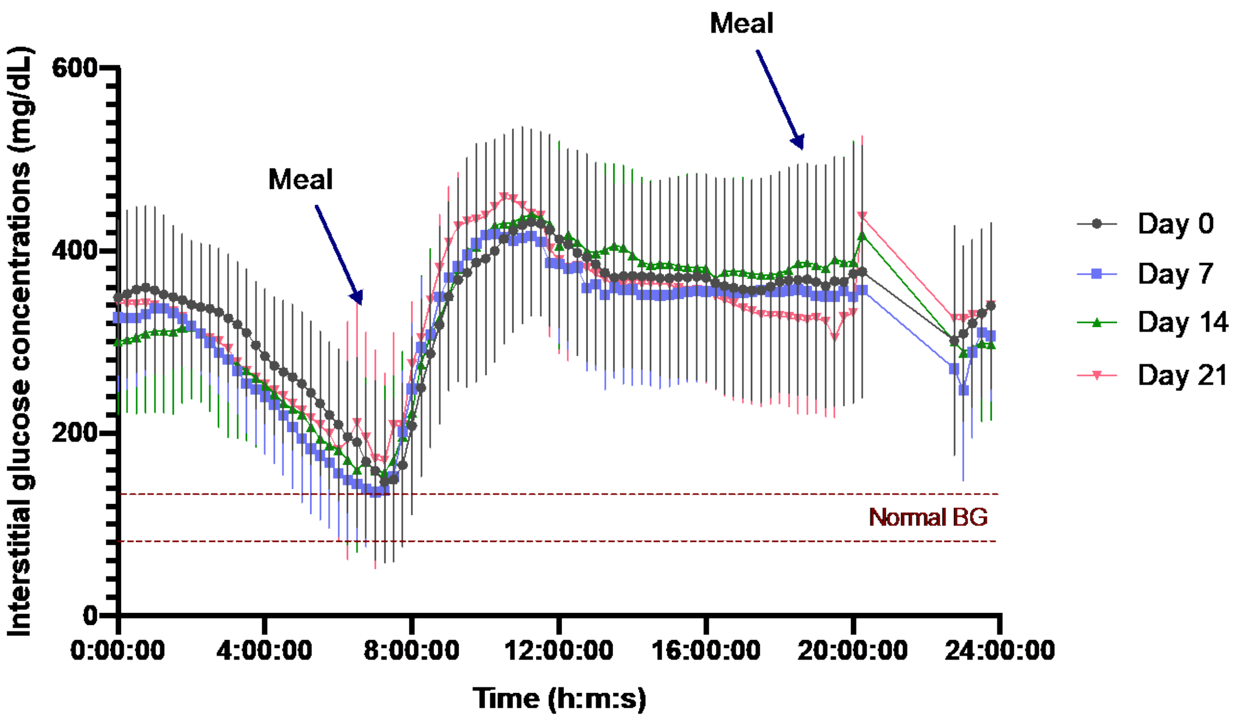

Fig. S1: Effects of 3 weeks fenofibrate administration on weekly interstitial glucose (IG) concentrations in diabetic dogs. Mean IG measurements over 24h following fenofibrate administration compared to pre-treatment values. All data represent the average from 7 dogs measured at 15-minute increments. Means ± SD are plotted.

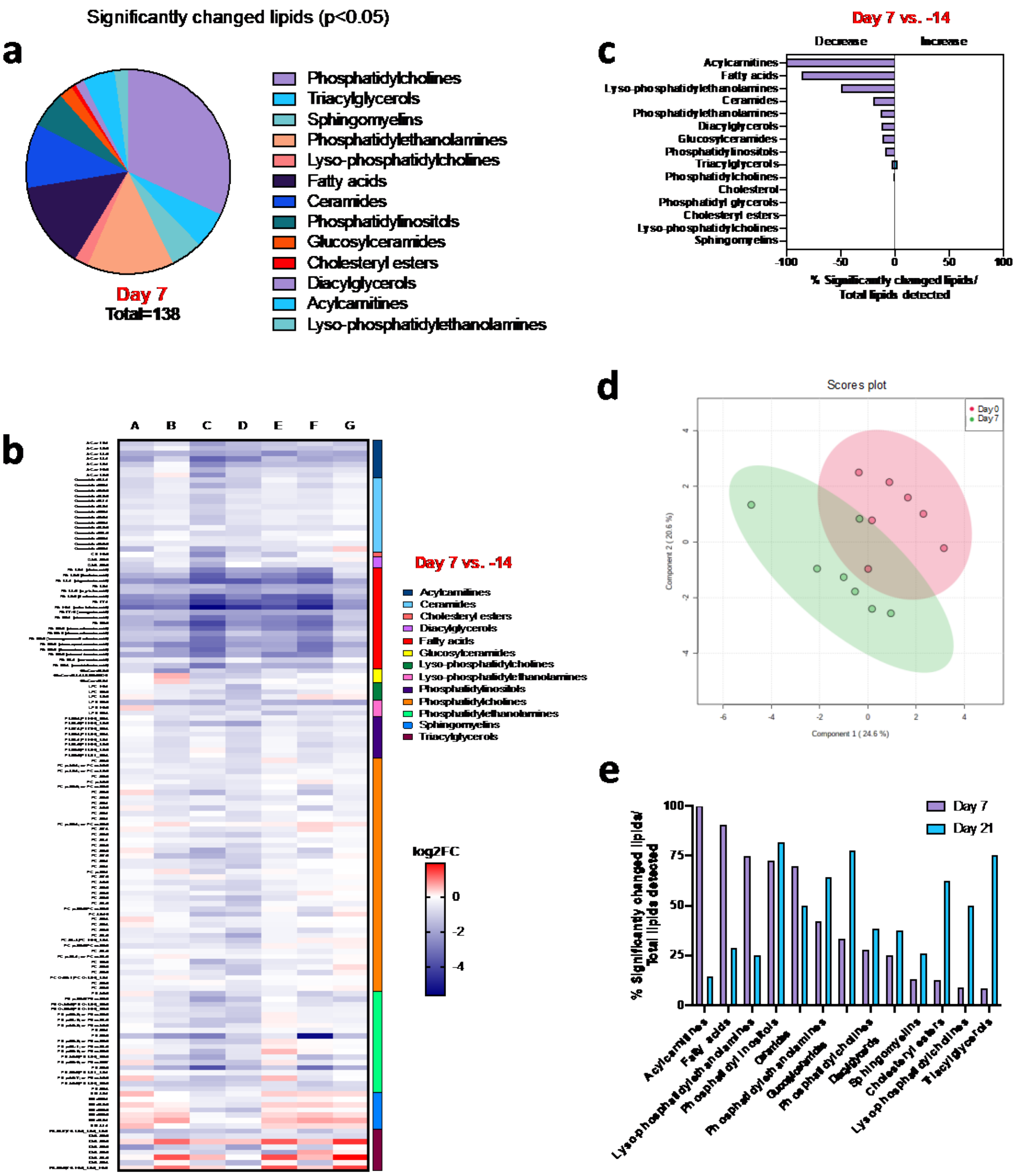

Fig. S2: Changes in the plasma lipidome after 1 week of fenofibrate treatment. (**a**) Composition of lipid groups altered after 1 week of fenofibrate treatment compared to baseline. Significance defined as $P < 0.05$. (**b**) Heatmap of all differentially abundant lipid species in plasma of diabetic dogs pre- and post-fenofibrate treatment. (*c*) Percentage of lipid groups that were differentially abundant after 1 week of fenofibrate administration. Significance was defined as $P < 0.05$ and fold-change > 1.5. (**d**) Principal component analysis of the plasma lipidome in diabetic dogs at baseline and 1 week after fenofibrate treatment. Circles represent regions of 95% confidence. (**e**) Comparison of lipid groups altered at 1 week and 3 weeks post-fenofibrate treatment.

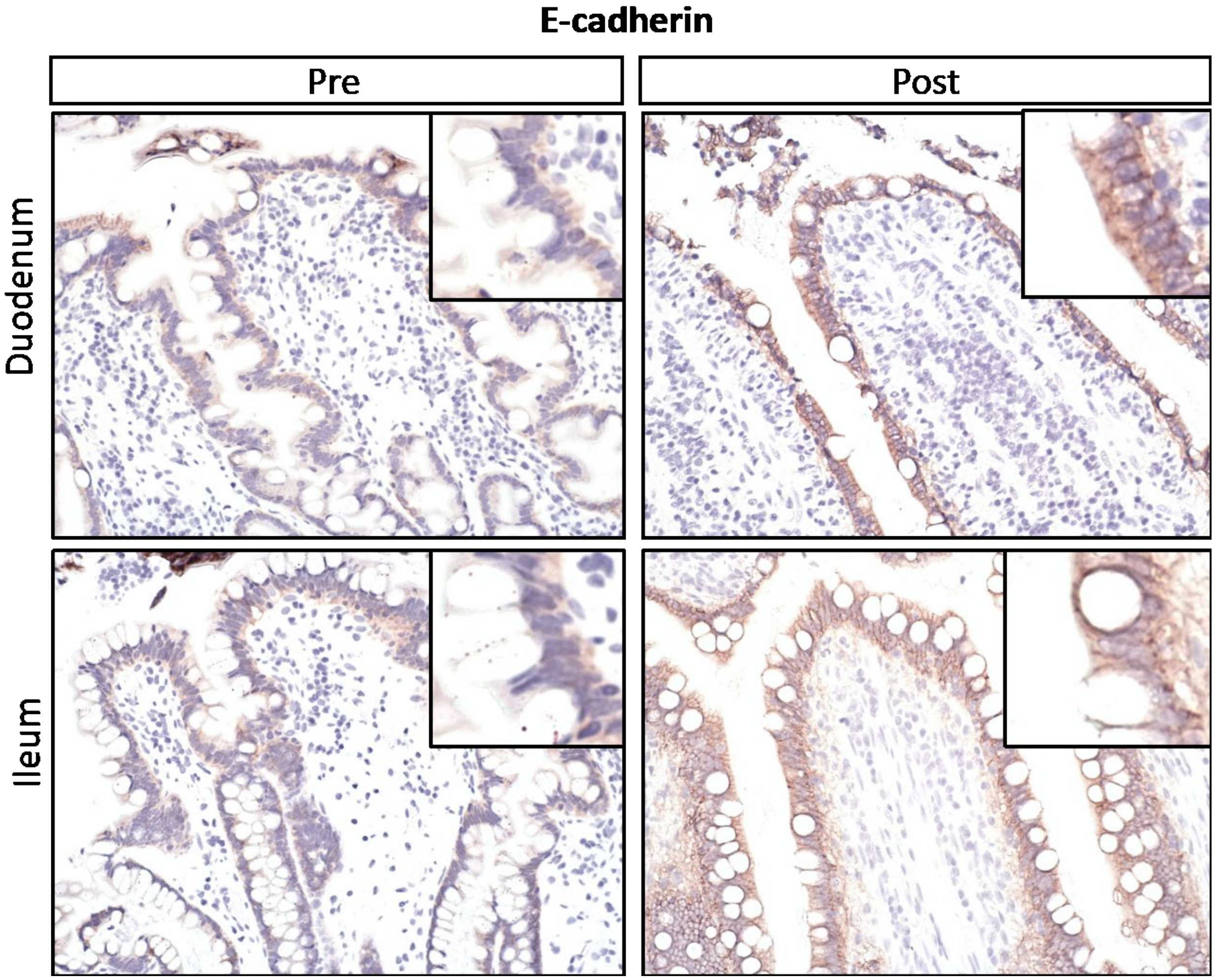

Fig. S3: Fenofibrate upregulates intestinal e-cadherin expression in diabetic dogs. Immunohistochemical staining of e-cadherin in the duodenum and ileum of diabetic dogs pre- and post-fenofibrate administration. Magnification: 40X

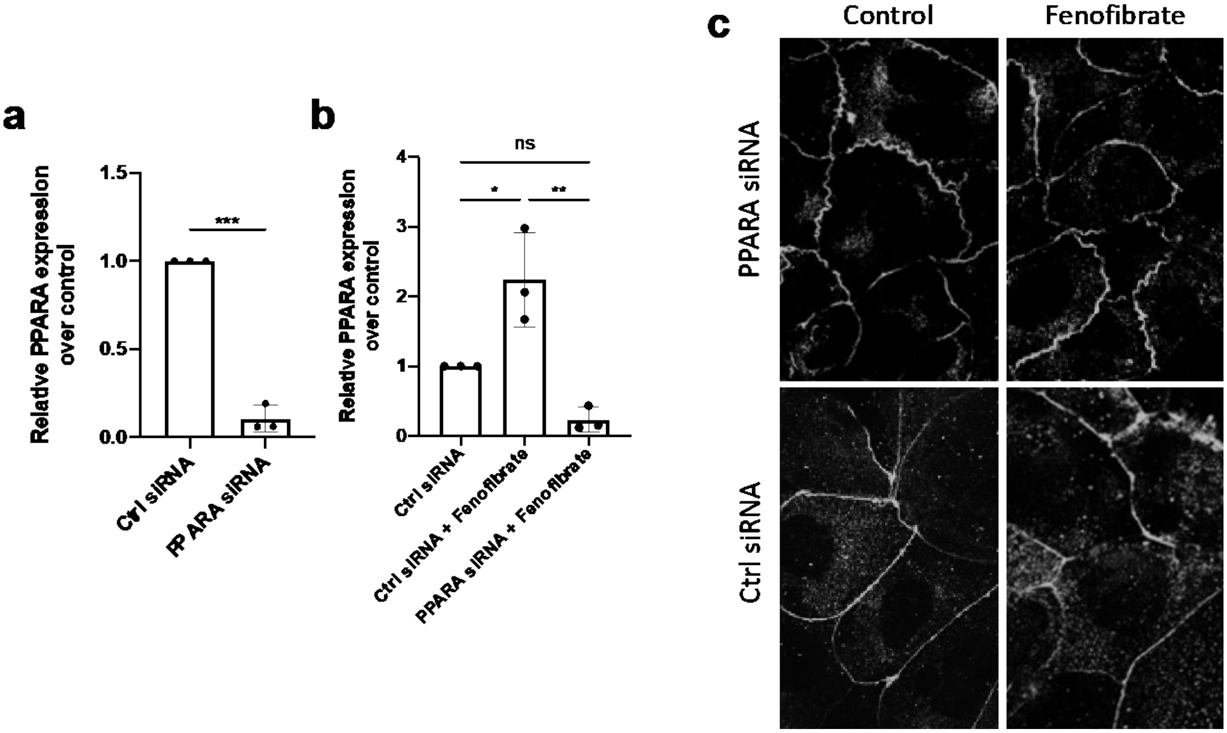

Fig. S4: PPARα expression is induced by fenofibrate. (**a**) Knockdown of PPARα in Caco-2 cells. (**b**) Expression of PPARα in Caco-2 cells transfected with PPARα siRNA following treatment with fenofibrate. (**c**) Presence of tight junction ZO-1 expression following PPARα knockdown and controls. *$P < 0.05$, **$P < 0.01$, ***$P < 0.001$ by one-way ANOVA with Tukey's multiple comparison test. Ns, not significant.

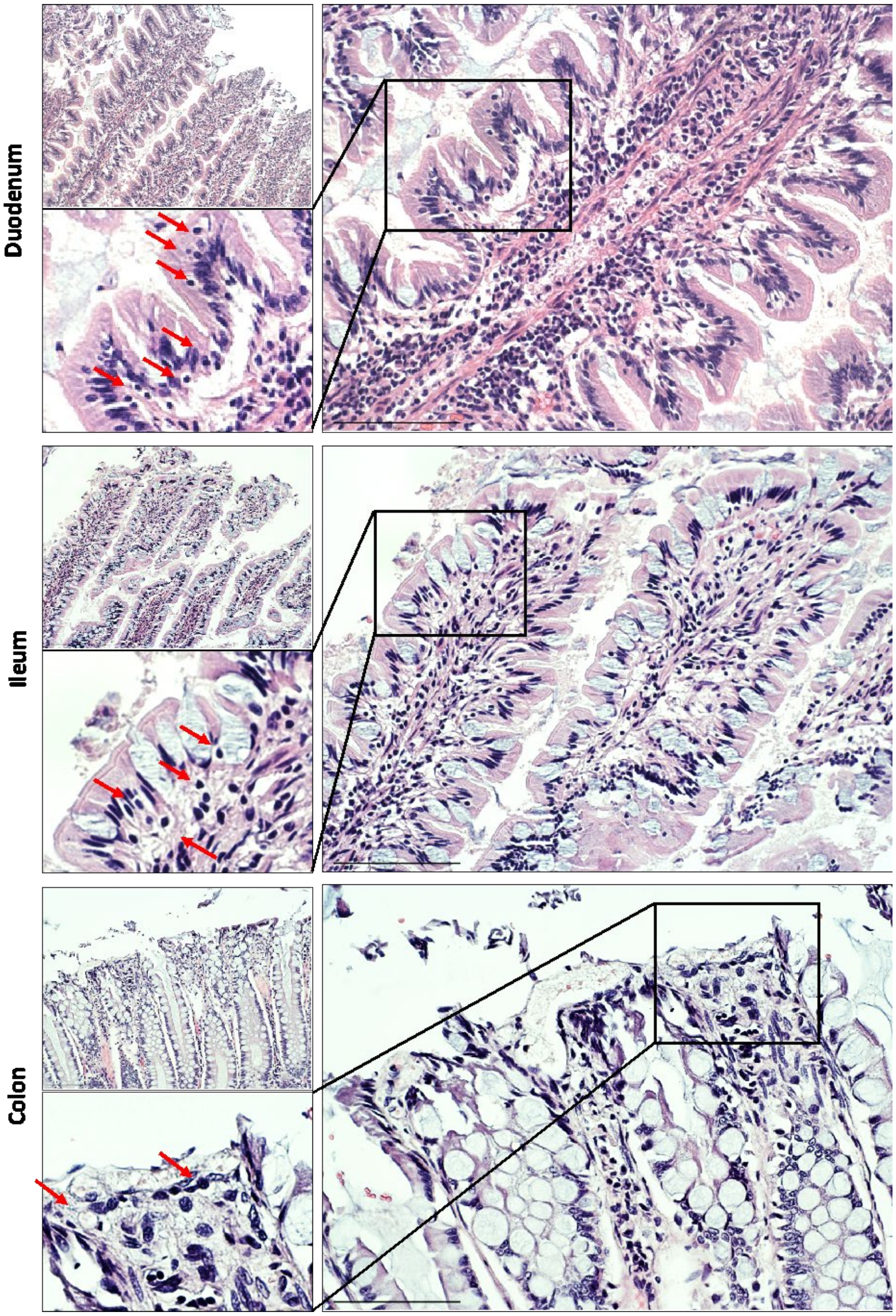

Fig. S5: Increased intraepithelial lymphocyte density in dogs with spontaneous diabetes. Lymphocytes (*red arrows*) in the intraepithelial compartments of duodenum, ileum, and colon. (H&E stain: *top left* 20X, *right* 40X*, bottom left* 100X)
